# Challenges Ahead for a Rational Analysis of Vitamin D in Athletes

**DOI:** 10.3389/fnut.2021.712335

**Published:** 2021-11-08

**Authors:** Pedro Araujo, Cioly Méndez-Dávila

**Affiliations:** ^1^Feed and Nutrition Group, Norwegian Institute of Marine Research, Bergen, Norway; ^2^Department of Rheumatology, University Hospital 12 de Octubre, Madrid, Spain

**Keywords:** vitamin D, sports, 25-hydroxyvitamin D, immunoassays, liquid chromatography mass spectrometry

## Abstract

Vitamin D is an essential vitamin for the normal formation of bones and calcium absorption. It is synthesized into our body through sunlight exposure and obtained by consuming foods rich in vitamin D (e.g., fatty fish, eggs yolk, dairy products). Its benefits on the health and performance of athletes are well documented. This article outlines some analytical challenges concerning the analytical quantification of vitamin D for its optimal intake, namely, a comprehensive study of the variability of the assay before categorizing any method as the golden standard, assurance of sample comparability to draw meaningful correlations, revision of the intake guidance based on appropriate statistical power analysis, and the implementation of rational strategies for preventing the underlying mechanism of preanalytical factors. Addressing these challenges will enable the effective management of vitamin D in the sports sector.

## Introduction

The benefits of sun exposure have been recognized since ancient times by different civilizations and cultures. In Ancient Greece, sunbathing was recommended to athletes participating in the Ancient Olympic Games (776 BC−393 AD) to perform well in their sport disciplines and for the beneficial power of sunlight on health (1–3). Nowadays, these observations are rationalized as the result of the ultraviolet radiation from sunlight striking the skin of the athletes and triggering vitamin D3 (cholecalciferol) synthesis, which has been associated with athletic performance and promotes calcium absorption and enables the formation and maintenance of strong bones (4). The use of artificial ultraviolet radiation was discussed in Germany and Russia by the end of the 1920s and 1930s as an aid to enhance the athletic performance of swimmers and sprinters, respectively (5, 6).

Exposure to natural or artificial ultraviolet radiation is not the only way to obtain vitamin D. The human body can also obtain vitamin D from food and supplements, such as biologically inert vitamin D2 (ergocalciferol from plant sources) and vitamin D3 (cholecalciferol from animal sources) which are converted to 25(OH)D (aka calcifediol or calcidiol) and biologically active 1,25(OH)2D (aka calcitriol) (7). It has been demonstrated that 25(OH)D is the main circulating form of vitamin D in the blood and the best indicator of vitamin D status (8) that is generally quantified by different analytical techniques, e.g., competitive protein binding assay, radioimmunoassay, enzyme-linked immunosorbent assay, high-performance liquid chromatography (HPLC), and liquid chromatography-tandem mass spectrometry (LC-MS/MS) to be discussed below.

**Figure 1:**
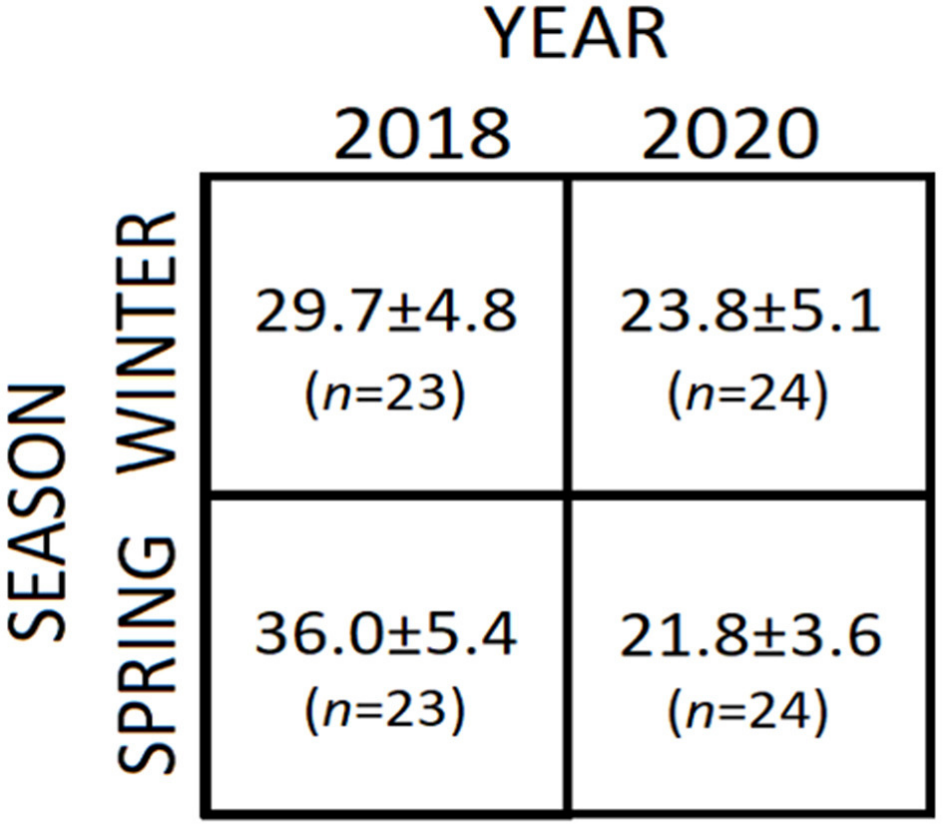
Chronological and seasonal variations of 25(OH)D in the serum from professional football players were published elsewhere (18). The values represent the means ± standard deviations (in ng/ml) and the number of participants (*n*).

Vitamin D deficiency is common in athletes and most reviews have demonstrated consistently that increasing serum 25(OH)D levels have a beneficial effect on muscle strength, power, and mass of the general population (9), and the muscle strength performance of athletes (10, 11). Modern athletes are aware that vitamin D deficiency might have a negative effect on their performance. It has been demonstrated, that the lack of vitamin D was clearly associated with the increased chance of muscle injuries in football players. In addition, a positive correlation between increased levels of vitamin D and injury prevention and recovery has been observed (12). Studies on indoor athletes from different disciplines (e.g., basketball, gymnastics) have observed a high prevalence (over 80%) of deficient levels of vitamin D (12–14). The same percentage (81%) was observed in outdoor athletes (e.g., football) who were categorized as vitamin D deficient (15). Similar trends have been reported in a meta-analysis study where a prominent 56% of the athletes from different nations and a wide range of indoor and outdoor disciplines were categorized as having inadequate levels of vitamin D (16). However, some studies have found no correlation between vitamin D and the performance of athletes which in turn was ascribed to small sample sizes (17). In addition to the deficiency of vitamin D, the current coronavirus disease 2019 (COVID-19) pandemic represents an additional burden and source of distress for athletes. Currently, there is no comprehensive cross-athletic comparison of vitamin D status and COVID-19; however, seasonal and chronological variations of the levels of circulating 25(OH)D in the serum from Japanese professional football players have been recently compared (Figure 1) and indicated, as expected, a significant seasonal increase of 21.2% between winter and spring 2018 (18). In contrast, the equivalent seasonal comparison for 2020 revealed a 25(OH)D reduction of 8.4%, which correlates with the restriction of outdoor training from February 8 onward by the Hong Kong Sports Institute (19). Figure 1 shows that between 2018 and 2020, there was a statistically significant reduction of 19.9% in winter and 39.4% in spring, indicating effectively that the ongoing COVID-19 pandemic has a negative impact on the vitamin D status of athletes.

Athletes who have been restricted from outdoor training to avoid/control the infection/transmission of COVID-19 will be at more risk of injuries in the aftermath of the pandemic because vitamin D levels are endogenously synthesized in response to sun exposure. Physicians from the sport governing committees should raise awareness on the importance of maintaining an appropriate intake of vitamin D to avert sports-related injuries. In addition, these observations highlight the need of establishing reliable cut-off values for giving safe intake guidance to athletes.

The beneficial health effects of vitamin D on athletes are widely discussed by the scientific and general community through national and international articles, forums, conferences, discussion panels, press, blogs, etc. Despite this great deal of attention, there are still some pending challenges that need to be addressed for the effective management of vitamin D, especially in the sports sector, where the importance of vitamin D has been recognized to have an impact on athletic performance. An overview of the current literature on vitamin D in connection with the sports sector has been performed and relevant articles are discussed to highlight some controversial (and sometimes ignored) analytical aspects of this important vitamin.

## Materials and Methods

To highlight the challenges ahead, the present manuscript reviewed the current body of evidence related to the controversial analytical aspects of vitamin D in the sports sector, frequently omitted in published studies, by using some examples from various literature. The search for articles was carried out between May and December 2020 using different databases (e.g., PubMed, ScienceDirect, Web of Science). The descriptors used in the context of vitamin D and/or sports were assay variability, sample/assay comparability, intake ranges, statistical power, and preanalytical factors.

The quality of the records was assessed by using the critical appraisal checklist proposed by the Joanna Briggs Institute (https://jbi.global) for documents that are focused on six questions, namely: (i) is the source of the opinion clearly identified?; (ii) Does the source of opinion have a standing in the field of expertise?; (iii) Are the interests of the relevant population the central focus of the opinion?; (iv) Is the stated position the result of an analytical process, and is there logic in the opinion expressed?; v) Is there reference to the extant literature?; (vi) Is any incongruence with the literature/sources logically defended? In addition, the current impact factor (when available) of the journals where the records were published was checked as a putative measure of quality.

## Results

The search identified 49 relevant articles that were reduced to 27 after removing the duplicate articles and records that did not comply with the critical appraisal checklist. The 27 articles in connection with vitamin D in the sports sector were read in full, discussed comprehensively, and the data from some selected articles were presented as graphics. The 27 relevant studies were organized into five controversial topics, more specifically, assay variability (20–28), sample/assay comparability (8, 9, 12, 27, 29, 30), intake ranges (12, 20, 31–34), statistical power (12, 35–38), and preanalytical factors (28, 39–43), that were comprehensively discussed. Out of all the records, 33% were used to discuss assay variability; 22% for sample/assay comparability, intake ranges, and preanalytical factors; and 19% for statistical power. A qualitative checking of the 27 records indicated that 37, 40.7, and 3.7% of the analyzed references were published in peer-reviewed journals with current impact factors ranging between 5–8.6 (8, 9, 22, 23, 30, 31, 33–35, 39), 2.4–4. (12, 21, 27–29, 32, 36, 38, 40, 41, 43), and 1.2 (39), respectively. There was one article, representing 3.7% of the records, that was published in a peer-reviewed journal of the Norwegian Medical Association without impact factor (37). Out of all the records, 14.8% from two well-reputed international organizations and a chemical supplier, namely, the European Food Safety Authority (EFSA), the Vitamin D External Quality Assessment Scheme (DEQAS), and Sigma-Aldrich were classified as scientific and technical reports (20, 25, 26, 42). The results are presented in narrative and graphical form.

## Discussion

The current state of knowledge on vitamin D has helped to understand many aspects of this important biomarker (e.g., production, sources, physiological effects). However, there are still some gaps that have brought a great deal of discussion in the scientific community and need to be worked through.

### Assay Variability

**Figure 2:**
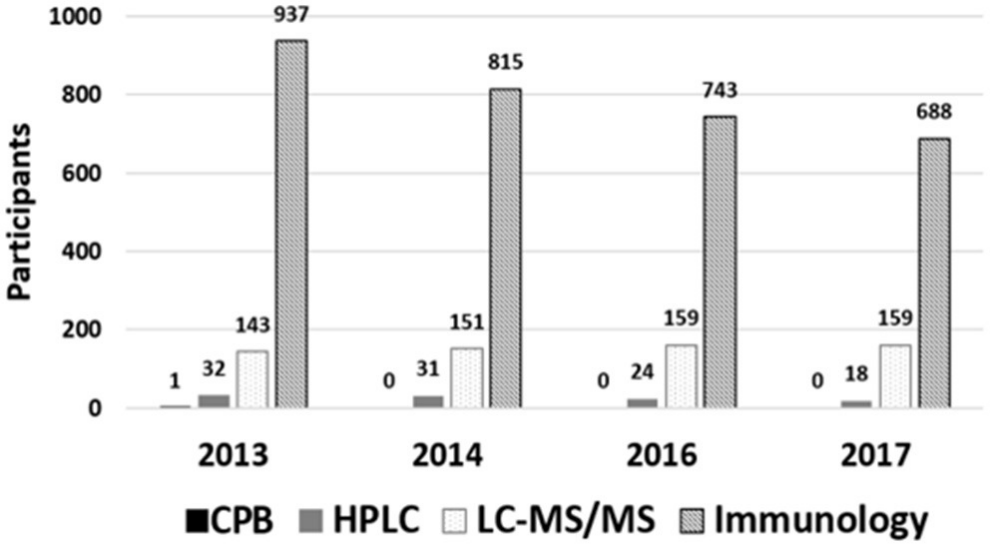
Methods used in the largest proficiency testing scheme for vitamin D analysis (25, 26).

For over four decades, the determination of the circulating 25(OH)D has been carried out using different methods and its pros and cons have been the subject of several publications (20, 21). Chronologically, the first method used for measuring 25(OH)D in the plasma was competitive protein binding (CPB) assay (22, 23). Eventually, radioimmunoassay (RIA) and enzyme-linked immunosorbent assay (ELISA) replaced the CPB method and rapidly became the preferred methods of reference laboratories and are often cited in different research studies. HPLC and LC-MS/MS have been also used for determining 25(OH)D successfully. Nowadays, LC-MS/MS is commonly referred to as the golden standard method for determining vitamin D status (24). Despite this powerful and attractive designation, it is undeniable that immunoassay methods are consistently the preferred assays among laboratories as reflected by the International Vitamin D Quality Assessment Scheme (DEQAS), which is the largest proficiency testing scheme for vitamin D. A summary of the different reports published by DEQAS (Figure 2) shows that between 2013 and 2017, the CPB method has not been considered by any participant (25, 26); the use of high-performance liquid chromatography (HPLC) has declined by 44% between 2013 and 2017; the LC-MS/MS methods exhibited a steady increase around 5% between 2013 and 2016, but it remained constant between 2016 and 2017; the immunoassay methods were the most popular among participants. However, despite the observed popularity, their use has decreased by 27% between 2013 and 2017.

Regarding the performance of the different methodologies for determining vitamin D, the DEQAS report has indicated that the variability, expressed as the ratio of the SD to the mean (*aka* coefficient of variation or CV), for the most used immunoassay (DiaSorin Liaison TOTAL, CV = 8.1%) and LC-MS/MS (CV = 9.4%) was comparable to the 10% threshold proposed by the Vitamin D Standardization Program (VDSP). Interestingly, a comparison study between the popular DiaSorin Liaison TOTAL and LC-MS/MS for determining the levels of 25(OH)D in serums has observed substantial variations (around 30%) in both assays and for repeated measurements at the same laboratory (27). The three times larger disagreement between this comparative study (27) and the International Vitamin D Quality Assessment Scheme (25, 26) indicates that neither LC-MS/MS nor immunoassays can be regarded as the golden standard methods for estimating the reference ranges for serums circulating 25(OH)D. It is undeniable that assay variability is an important challenge that must be addressed comprehensively and rationally before labeling any technique as the golden standard method. Some authors have emphatically pinpointed that the variability between assays is responsible for the limited progress toward the establishment of reference values for 25(OH)D in health (28). Furthermore, the criteria used to categorize a method as the golden standard for vitamin D should be re-evaluated before validating the official or unofficial reference ranges.

### Sample and Assay Comparability

The involvement of vitamin D in athletic performance has been discussed in research and review articles, where the circulating 25(OH)D levels in athletes from various sports disciplines were measured by different assays (9, 12, 29). The main drawbacks of some reviews are that the levels of 25(OH)D obtained by different assays are compared without acknowledging the inherent discrepancy in variability associated with the biological specimens and/or the variability associated with the different assays. For example, blood samples from athletes were taken to produce serum or plasma, and the processes behind their production yield specimens with different matrices. Plasma preparation involves the addition of exogenous agents (e.g., anticoagulants), removal of cellular components, and the presence of coagulation factors (e.g., fibrinogen), while serum preparation involves a coagulation process, the presence of cellular components, and the absence of coagulation factors. Although the differences in the matrices of the samples and the implemented assays might significantly affect the vitamin D results, these factors are rarely considered in comparative studies. For example, some researchers have discussed the vitamin D dosage for optimal athletic performance in the context of the levels proposed by the Institute of Medicine (IOM) by comparing seven groups of athletes whose levels of 25(OH)D in the serum (six groups) and plasma (one group) were determined by four different assays (ELISA, RIA, chemiluminescence immunoassay (CLIA) and LC-MS/MS) (9). It has been reported that the 25(OH)D in plasma is not a reliable biomarker of vitamin D status (8, 30). In addition, some studies concerned with the determination of 25(OH)D in serums by different assays have reported dramatic differences between LC-MS/MS, RIA, and CLIA at a cut-off of 20 nmol/L (insufficient) (27). In this specific study, a remarkable lack of agreement between the different analytical methods was observed (27). For instance, the reported 25(OH)D mean values in serums by LC-MS/MS, RIA, and CLIA were 34, 28, and 24 ng/ml, respectively. In addition, the proportions of 8, 22, and 43% of participants were classified as vitamin D insufficient when LC-MS/MS, RIA, and CLIA assays were used to measure 25(OH)D, respectively. The present article is not judging the reliability of the conclusions derived from the comparison of the seven groups of athletes (9), but it is highlighting the importance of discussing vitamin D dosage for optimal athletic performance based on similar techniques and biological samples to ensure comparability and to draw meaningful conclusions.

### Ranges for Vitamin D

The benefits of adequate vitamin D levels in athletes and its impact on health and performance have been published elsewhere (12). Some cohort studies on the effect of vitamin D supplementation and further measurement of its metabolite 25(OH)D have based their discussions and conclusions either on the recommended ranges by competent authorities, such as IOM or the European Food Safety Authority (EFSA) (20) or on the proposed ranges by well-reputed experts on vitamin D (31). The various recommendations have created a long-standing controversy among vitamin D researchers over the appropriate reference ranges, hence an open debate between the different key players in the field of vitamin D is required urgently to adopt a common range or perhaps several ranges if vitamin D status is associated with specific cohort (e.g., race/ethnicity, sex).

**Figure 3:**
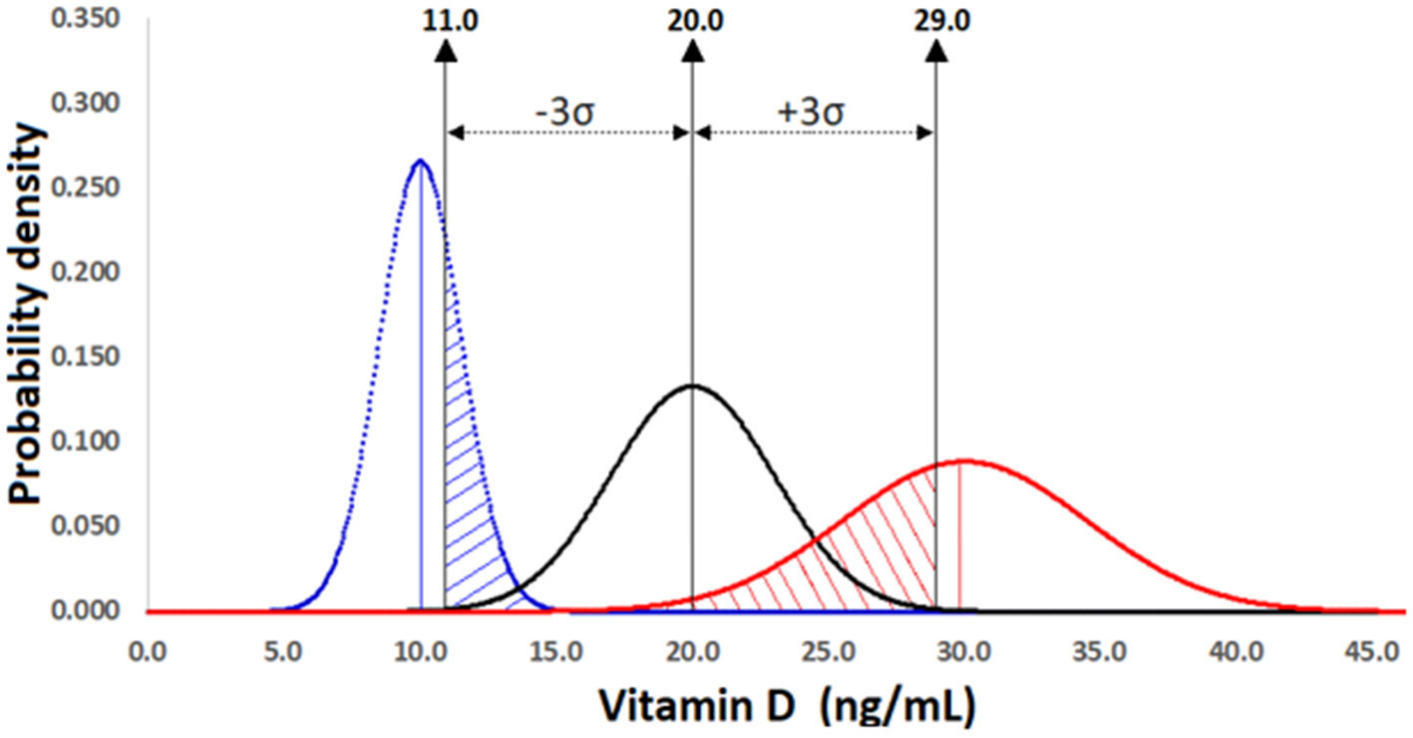
Comparison of three normally distributed populations of athletes with 25(OH)D mean levels of 10 ng/ml (insufficient, blue line curve), 20 ng/ml (deficient, black line curve), and 30 ng/ml (sufficient, red line curve) and the same coefficient of variation (CV = 15%). An overlapping of 0.25 (oblique blue and red lines) is observed between insufficient-deficient and sufficient-deficient within the range ±3σ of the deficient population, which causes a statistical power of 0.75 lower than the minimum desirable of 0.80. The mean values were obtained from a study on athletes published elsewhere (34).

A recent article collected data, between 2006 and 2017, from 5,000,000 patients with different levels of 25(OH)D to establish their status according to the range proposed by IOM (32). This 10-year data revealed a general decrease in the frequency of patients labeled as deficient (<10 ng/ml) and insufficient (10–24 ng/ml) and a general increase in those labeled as sufficient (25–80 ng/ml) and toxic (>80 ng/ml). However, assay variation is a potential factor that might confound the diagnosis (33). The following example demonstrates that a normally distributed population with a mean value of 20 ng/ml of vitamin D in serums [regarded as insufficient for athletes (34)] and a CV of 15% [an accepted CV for immunoassay of 25(OH)D] exhibits a considerable overlapping with two normally distributed populations with the mean values of 10 and 30 ng/ml of vitamin D [regarded as deficient and sufficient, respectively (34)] and with the same CV of 15% (Figure 3).

### Statistical Power

The substantial percentages of the overlapping areas in Figure 3, namely, insufficient-deficient (blue oblique lines) and sufficient-deficient (red oblique lines) indicate that the inherent variability of a method will affect the decision on adequate or poor vitamin D status. Assay variability is a potential confounding factor that is often overlooked in studies on the association between vitamin D and athletic performance as deducted from the apparent lack of benefit of 25(OH)D at levels above 50 ng/ml in the skeletal muscle of athletes (12), a level considered as sufficient.

The observations from the previous sections highlight the importance of understanding the analytical aspects of the methods currently used for the quantitative determination of vitamin D and the incorporation of appropriate statistical analyses to support the experimental results.

Figure 3 shows that a remarkable portion of the insufficient curve (mean 10 ng/ml and CV = 15%), more specifically, 0.25 of the total standardized area is confounded with the deficient curve (mean 20 ng/ml and CV = 15%). Therefore, the statistical power or confidence with which it is possible to detect a difference if one exists is represented by the area between 5 and 11 ng/ml and will be 0.75 (1.00–0.25 = 0.75). Although there is not a conventional criterion to determine what is a suitable statistical power, a value of 0.80 is generally considered the minimum desirable. A similar overlapping (Type II error) of 0.25 and statistical power of 0.75 (area between 29 and 43.5 ng/ml) were obtained when the sufficient (30 ng/ml and CV = 15%) and deficient groups were compared. The results of this example suggest that an assay with an inherent variability lower than 15% is required to achieve the minimum statistical power of 80% between the categories insufficient-deficient and sufficient-deficient. It is evident from Figure 3 that the distinction between insufficient (blue distribution) and sufficient (red distribution) is characterized by a statistical power of 1. It is important to mention that in addition to the assay variability and confidence level, the sample size (number of participants) might have an impact on the statistical power of a study.

The confidence level is widely reported in the literature; however, the statistical power is not very often acknowledged in vitamin D studies (35). The reasons for the omission of the term 1–β in the general comparison studies have been ascribed to the difficulties associated with its quantification and the cursory treatment of the subject in statistical books (36). In addition, some authors have pinpointed that despite the impressive size of some randomized control trials on vitamin D, their statistical power is insufficient to rule out the lack of effect (35, 37, 38), indicating that the relationship between sample size and power is not linear and that statistics should be always treated with caution. Further studies on vitamin D should try to include not only the confidence level but also the statistical power. A lack of compliance with this premise might have serious implications in the categorization of an individual in a particular vitamin D range.

### Preanalytical Factors

The analysis of vitamin D in different studies and national surveys, using different kinds of analytical approaches and further within/between comparisons of methods and laboratories, have significantly improved the harmonization of the different analytical techniques and the quality of the results. However, it is equally important to understand and reduce the impact of preanalytical factors on vitamin D analysis. There are multiple preanalytical factors that might affect the stability of vitamin D (e.g., light, temperature, storage conditions, collection devices). However, it is surprising to note the paucity of information on vitamin D and its preanalytical factors.

Although the quantification of the circulating concentration of 25(OH)D is the only available approach to assess the vitamin status in humans, there are still some intrinsic difficulties associated with the nature of vitamin D and the biological matrix that have hindered the development of reliable assays. On the one hand, the lack of polar groups in the structure of vitamin D that allows its transportation in blood by the vitamin D-binding protein (DBP) has been identified as a potential source of variability for both manual and automated immunoassays due to the incomplete release of 25(OH)D from the DBP resulting in reduced sensitivity (39, 40). On the other hand, whole blood, and its derived specimens (plasma and serum) are regarded as one of the most complex biological matrices that might negatively affect the immunoassays and chromatographic-based methods. It has been demonstrated that blood viscosity alters the binding efficiency and specificity for immunoassay detection (41); that the presence of vitamin D2, vitamin D3, and multiple vitamin D metabolites in serum and plasma can exhibit cross-reactivity or coelution in immunoassays or chromatographic methods (28); and that the coelution of phospholipids along with vitamin D might cause serious sensitivity and reproducibility issues resulting in irregularities in quantitation (42).

It is vital to adopt strategies for understanding and preventing the underlying mechanisms of preanalytical factors with larger sample sizes. Besides, analytical and post-analytical factors must also be considered to determine robust reference values, define more precisely the status of vitamin D (43), and uncover its associations with athletic performance.

## Conclusions

Vitamin D has been recognized for having an impact on athletic performance and its effective management in the sports sector is a pending challenge that should be addressed appropriately. Considering the status quo and the vulnerability of athletes to be exposed to inappropriate doses of vitamin D, it is advisable to follow the recommendations of health care professionals to avoid the detrimental effects (e.g., injuries, illness) associated with an incorrect supplementation of vitamin D. It is also important to encourage researchers to use appropriate statistical tools and optimal sample sizes to assure adequate power to detect statistical significance, to draw robust conclusions, and to propose reliable reference ranges for vitamin D. Scientific journals can play an important role in this respect by promoting the implementation of statistical power analysis and requiring, wherever possible, the power estimates from articles which might have implications on human health.

## Author Contributions

PA conceived the study, performed the statistical analysis, and wrote the first draft of the paper. CM-D contributed to the design and revised the manuscript for important contents. PA and CM-D conducted the literature search, literature screening, and extracted the data. PA and CM-D read the article and approved the final version. All authors contributed to the article and approved the submitted version.

## Funding

This study was supported by the Institute of Marine Research (IMR), Bergen, Norway.

## Conflict of Interest

The authors declare that the research was conducted in the absence of any commercial or financial relationships that could be construed as a potential conflict of interest.

## Publisher's Note

All claims expressed in this article are solely those of the authors and do not necessarily represent those of their affiliated organizations, or those of the publisher, the editors and the reviewers. Any product that may be evaluated in this article, or claim that may be made by its manufacturer, is not guaranteed or endorsed by the publisher.
